# Meprin β activity modulates cellular proliferation via trans-signaling IL-6-mediated AKT/ERK pathway in IR-induced kidney injury

**DOI:** 10.21203/rs.3.rs-5901359/v1

**Published:** 2025-01-30

**Authors:** Shaymaa Abousaad, Faihaa Ahmed, Ayman Abouzeid, Christine Adhiambo, Elimelda Ongeri

**Affiliations:** North Carolina A&T State University; the College of Veterinary Medicine, North Carolina State University; North Carolina A&T State University; North Carolina A&T State University; North Carolina A&T State University

**Keywords:** interleukin-6, proliferation, ischemia-reperfusion, meprin metalloprotease β

## Abstract

Inflammation plays a central role in the progression of kidney injury induced by ischemia/reperfusion (IR). Meprin metalloproteinases have been implicated in the pathophysiology of IR-induced kidney injury. Existing data from in vitro and in vivo studies show that meprins modulate interleukin-6 (IL-6)-mediated inflammation via proteolytic processing of IL-6 and its receptor. IL-6 trans-signaling induces proliferation through either MAPK/ERK or PI3K/AKT pathway or in crosstalk with AKT/ERK. We previously showed that meprin β modulates cellular survival (BCL-2) through IL-6/JAK/STAT signaling pathway in IR-induced kidney injury. However, it’s not known how meprin β modulation of the IL-6 signaling pathway impacts the cellular proliferation in IR-induced acute kidney injury. The goal of the current study was to determine how meprin β modulation of the IL-6 signaling pathway impacts downstream cellular proliferation in IR-induced kidney injury. We used the unilateral IR as a model of renal inflammation in wild-type (WT) and meprin β knockout (βKO) mice, with the contralateral kidneys serving as controls. The mice were sacrificed at 96 h post-IR, and kidney tissue processed for evaluation by RT-PCR and immunohistochemistry. Statistical analysis utilized two-way ANOVA. RT-PCR data showed a significant increase in mRNA levels for IL-6 and proliferating cell nuclear antigen (PCNA) in WT and βKO mice at 96 h-post IR when compared to WT control kidneys. However, the baseline mRNA levels for PCNA were significantly higher in βKO when compared to WT kidneys. Immunohistochemical data showed significant increases in IL-6, PCNA, p-AKT and p-ERK in select tubules in both genotypes at 96 h post-IR when compared to control kidneys for each genotype. Data from immunofluorescence counterstaining of kidney tissues revealed that at 96 hours post-IR, IL-6, PCNA, p-AKT, and p-ERK were primarily expressed in meprin β-expressing proximal tubules (PTs), where meprins are abundantly present. However, high levels of IL-6 were also present in the lumen of PTs and DTs from WT and βKO kidneys at 96 h post-IR, suggesting increased release/shedding into filtrate and subsequently into urine. In conclusion, this study highlights the role of meprin β activity in regulating cellular proliferation through PCNA regulation, driven by the IL-6-mediated AKT/ERK signaling pathway during the recovery phase following IR-induced kidney injury.

## Introduction

Ischemia/Reperfusion (IR) is one of the leading causes of acute kidney injury (AKI) and is associated with high morbidity and mortality rates ^1,2^. Inflammation contributes to the pathology of AKI ^3,4^, particularly, IR-induced kidney injury ^5–7^. At the onset of inflammation, monocytes are recruited to the injury site and differentiate into M1 macrophages, key players in the inflammatory response ^8^. These macrophages release and respond to IL-6, which signals through two pathways: the classic pathway via membrane-bound IL-6R (mbIL-6R), predominantly on immune cells, and the trans-signaling pathway via soluble IL-6R (sIL-6R), enabling IL-6 activity in cells lacking mbIL-6R, such as renal proximal tubule epithelial cells ^9,10^. IL-6 trans-signaling plays a protective role in repair processes, particularly in ischemia-reperfusion-induced AKI models. Meprins metalloproteases, abundant in brush-border membranes of proximal kidney tubules ^11^, modulate inflammation by inactivating IL-6 and cleaving IL-6R, generating sIL-6R and influencing the balance between IL-6 pathways ^11–14^. Proliferating cell nuclear antigen (PCNA) is a well-known cellular proliferation marker that is induced through activation of IL-6 trans-signaling ^15–17^. When IL-6 binds to its receptor, it forms the IL-6/IL6R complex, which binds to the membrane-bound gp130 dimer, leading to activation phosphorylation of Janus Kinase- Signal Transducer and Activator of Transcription (JAK/STAT), Mitogen-activated protein kinase (MAPK) and phosphatidylinositol 3-kinase/Akt kinase (PI3K/AKT) pathways ^18^. Activation of these pathways modulates the expression of different inflammation related downstream pathways, such as cellular apoptosis, survival and proliferation ^19^. We previously showed that meprin β modulates cellular apoptosis and survival through IL-6/JAK/STAT signaling pathway in IR-induced kidney injury ^20^. However, proliferation was reported to be induced via trans-signaling IL-6 through MAPK/ERK signaling pathway ^21,22^, PI3K/AKT pathway ^23–25^ or their crosstalk (AKT/ERK) ^26–29^. It’s not known which of these mechanisms is involved in inducing the cellular proliferation when meprin β regulates the trans-signaling IL-6. Therefore, the goal of the current study was to determine how meprin β activity impact IL-6 mediated ERK/AKT pathway and downstream cellular proliferation in IR-induced kidney injury.

## Materials and methods

### Experimental animals

Twelve-week-old male mice Wild-type (WT) and meprin β knockout (βKO) male mice on a C57BL/6 background were used. The WT mice express both meprin A and meprin B, while the βKO mice are deficient in meprin B (β-β) and the heterodimeric form of meprin A (α-β). The βKO mice were generated by the laboratory of Judith Bond, Pennsylvania State University. The βKO mice were bred at the Laboratory Animal Resource Unit (LARU), North Carolina A&T State University (NC A&T). Age-matched WT mice were purchased from Charles River Laboratories (Wilmington, MA). The mice were housed in groups of up to five mice per standard cage and were fed a standard rat chow (Purina, St Louis MO) and water ad libitum with exposure to a 12:12 hour light-dark cycle. All the animal protocols for this study were approved by the NC A&T Institutional Animal Care and Use Committee (IACUC).

### Surgical induction of ischemia/reperfusion

Surgical procedures were used to induce ischemia/reperfusion on 12-week-old male mice. In summary, this was achieved by clamping the renal pedicle of the kidney for 27 minutes as previously described ^20^ followed by 96 h reperfusion. The contralateral kidney was not clamped and served as the control for each mouse. The mice were then euthanized by CO2 asphyxiations and kidney tissues harvested for proteomics and immunohistochemical analysis.

### Processing of kidney tissues

The harvested kidneys were de-capsulated, and sections of each kidney processed appropriately for RNA extraction, and paraffin embedding and subsequent immunohistochemistry. For immunohistochemistry, 2 mm mid-section tissue samples were stored in Carnoy’s fixative (60% ethanol/30% chloroform/10% acetic acid) overnight at 4°C, then transferred to 70% ethanol at 4°C until processed for paraffin embedding. Paraffin embedding and cutting tissue sections onto slides were performed at the Wake Forest University Pathology Laboratory. The kidney tissue samples for RNA extraction were stabilized and stored in RNALater^®^ solution (Invitrogen, Carlsbad, CA) for 24 hours at 4°C, then, the RNALater was aspirated, and tissues were stored at − 80°C until used for RT-PCR analysis.

### Assessment of kidney injury

**Figure 1:**
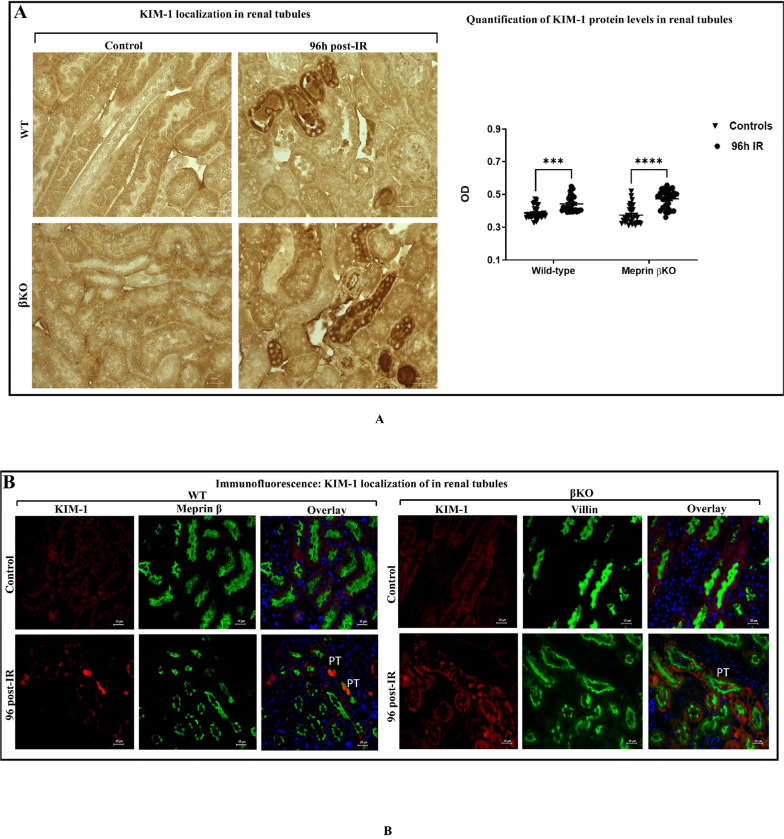
Kidney injury assessment in kidney tissue from wild type (WT) and meprin β deficient mice (βKO) at 96 h post-IR. Immunohistochemical staining for kidney injury marker 1 (KIM-1) in wild-type (WT) and meprin β knockout (βKO) kidneys at 96h post-IR. OD data were quantified using Image J analysis Software (ImageJ/Fiji 1.46) and analyzed by two-way ANOVA for 10 non-overlapping fields from renal tubular sections from each kidney **(A)**. Images at 60× magnification and the scale bar representing 20 μm. Immunofluorescence counterstaining of KIM-1 (red) and meprin β (green) in wild-type (WT) and villin (green) in meprin β knockout (βKO) kidneys to determine KIM-1 protein localization as an indicator of kidney injury in kidney tissues of both genotypes **(B)**. DAPI (blue) was used to stain the nuclei. Images at 60× magnification with a scale bar representing 20 μm. There was significant increase in KIM-1 in select tubules at 96 h post-IR in both genotypes confirming kidney injury. Data is expressed as mean ± SEM with P values as indicated, P ≤ 0.05 are considered statistically significant.

Since injury was not induced in the contralateral kidney, blood samples could not be used for biochemical assessment of kidney function. Instead, sections of each kidney were subjected to immunohistochemical staining for kidney injury molecule-1 (KIM-1), an established kidney injury biomarker. To confirm injury in kidneys subjected to IR, optic densitometry of the immunohistochemical staining was used to quantify the KIM-1 protein levels. Immunofluorescence was also used to determine the localization of the KIM-1. The data showed that there were significant increases in KIM-1 in select tubules at 96 h post-IR in both genotypes (p < 0.001) confirming that IR induced kidney injury (Fig. 1, panel A).

### RNA extraction and cDNA synthesis

Kidney tissues were disrupted using a tissue homogenizer (Bead Mill 4 Homogenizer, Thermo Scientific, Waltham, MA) and total RNA from control and ischemic kidneys were isolated using the Qiagen RNeasy Mini Kit (Qiagen, Germantown, MD) according to manufacturer’s guidelines. Concentration and purity were determined at 260/280 and 260/230 using a spectrophotometer (Spectrophotometer NanoDrop 2000, Thermo Scientific, Wilmington, DE). Denatured RNA was reverse transcribed into cDNA in a 20 μl reaction volume using High-Capacity cDNA Reverse Transcription Kit with RNase Inhibitor (ThermoFisher, Waltham, MA). Reverse transcription was performed at 37°C for 90 minutes, 85°C for 3 minutes, followed by quick chilling on 4°C and obtained cDNA stored at −20°C until subsequent amplification.

### Real-time PCR analysis

Two-steps RT-PCR reaction were performed with QuantiFast SYBR^®^ Green PCR Reagents (Qiagen, Germantown, MD) according to the manufacturer’s instructions using Bio-Rad Multiplate^™^ 96-Well PCR Plates as previously described ^20^. The qPCR cycling conditions were 50°C for 2 min, 95°C for 10 min followed by 40 cycles of a two-step amplification program (95°C for 15 s and 58°C for 1 min). At the end of the amplification, melting curve analysis was applied using the dissociation protocol from the Sequence Detection System to exclude contamination with non-specific PCR products. Oligonucleotides for all genes were designed as mouse-specific primer pairs obtained from Integrated DNA Technologies (IDTDNA) (Corlvielle, IO). Oligonucleotides sequences of the primer sets are: (i) IL-6, Forward: GTT CTC TGG GAA ATC GTG GA, Reverse: TGT ACT CCA GGT AGC TAT GG ^30^; (ii) PCNA, forward: GAC GCG GCG GCA TTA AAC, Reverse: GTT CAC GCC CAT GGC CAG; (iii) GAPDH, Forward: AGG TCG GTG TGA ACG GAT TTG, Reverse: GGG GTC GTT GAT GGC AAC A Data were analyzed via the 2^−ΔΔCt^ method ^31^ and mRNA expressions of target genes were presented as fold change relative to control samples of WT kidneys after normalization using the mRNA of housekeeping gene GAPDH of triplicate combinations from 4 mice per group ^32^.

### Immunohistological analysis

Immunohistochemical staining was used to evaluate the protein expression of IL-6, p-AKT, p-ERK1/2, and PCNA using previously described protocols ^20,33–35^. In summary, slides were deparaffinized by immersing in Xylene 2 times for 5 minutes each, 100% Ethanol 2 times for 3 minutes each, 95% Ethanol 2 times for 3 minutes each and distilled water 1 time for 5 minutes. Slides were then exposed to antigen unmasking via boiling in 10 mM sodium citrate buffer, pH 6.0, for 10 min. The slides were then immersed in methanol (MeOH) quench buffer (25% of 30% H_2_O_2_ in MeOH) for 20 minutes to quench endogenous peroxidase activity. Slides were washed for 5 minutes in PBS, then incubated in 1% normal goat serum in PBS buffer at room temperature for 1h in a humidified chamber to block the non-specific binding sites. Slides were then incubated in primary antibodies diluted in PBS buffer with 2.5% normal goat serum overnight at 4°C or at room temperature for 1 h. Antibodies used were; rabbit polyclonal anti-KIM-1 antibodies (Abcam Cat# ab47635, RRID:AB_882998) diluted 1:100, mouse monoclonal anti-IL-6 (Abcam Cat# ab9324, RRID:AB_307175) diluted 1:1000, rabbit monoclonal anti-PCNA (Cell Signaling Technology Cat# 13110, RRID:AB_2636979) diluted 1:4000, rabbit polyclonal anti-p-AKT (Cell Signaling Technology Cat# 9271, RRID:AB_329825) diluted 1:100, rabbit polyclonal anti-p-ERK1/2 (Cell Signaling Technology Cat# 4370, RRID:AB_2315112) diluted 1:200. After that, slides were washed in PBS 3 times for 5 minutes each. Slides were incubated for 30 minutes in a secondary antibody solution (PBS buffer with 2% universal biotinylated secondary antibody and 2% normal goat serum) and then washed in PBS 2 times for 5 minutes each. For standard immunostaining, we used the Vectastain^®^ Elite^®^ ABC Universal Kit Protocol (Vector Laboratories Cat# PK-6200, RRID: AB_2336826) following the manufacturer’s instruction. To determine staining intensity, the tissue sections were evaluated for IL-6, PCNA, p-AKT, and p-ERK1/2 levels using light microscope (KEYENCE Corporation of America, Elmwood, NJ) and imaged using BZ-X700 analysis Software. Ten non-overlapping fields for tubular and ten non-overlapping fields of renal corpuscle sections were imaged at 60 X magnification from each section of calibrated 8–bit images for optical density values (ODs) and quantified OD standard via Image J analysis Software (ImageJ/Fiji 1.46).

### Immunofluorescence staining

Immunofluorescence was used to determine the localization of the proteins of interest according to the previously described protocol with meprin β and villin as proximal tubule biomarkers for WT and βKO kidney tissue, respectively ^20,33^. Briefly, slides were deparaffinized by immersing in Xylene 3 times for 5 minutes each, 100% Ethanol 2 times for 10 minutes each, 95% Ethanol 2 times for 10 minutes each and distilled water 2 times for 5 minutes each. Slides were then exposed to antigen unmasking via boiling in 10 mM sodium citrate buffer, pH 6.0, for 10 min. Sequential immunofluorescence methods were performed according to the host of the secondary antibody species, and slides were incubated in a blocking buffer composed of PBS with either 5% normal goat serum, 5% chicken serum, or 5% donkey serum and 0.3% Triton X-100 for 30 minutes at room temperature to block non-specific binding sites. Slides were then incubated in primary antibodies diluted in a dilution buffer of PBS with 0.3% Triton X-100 and 1% BSA and incubated overnight at 4°C or at room temperature for 1 h. The primary antibodies included: goat anti-mouse meprin β polyclonal antibodies (R and D Systems Cat# AF3300, RRID: AB_2143451) diluted 1:200; mouse monoclonal anti-villin antibodies (Santa Cruz Biotechnology Cat# sc-58897, RRID: AB_2304475) diluted 1:200; rabbit polyclonal anti-KIM-1 antibodies (Abcam Cat# ab47635, RRID:AB_882998); mouse monoclonal anti-IL-6 (Abcam Cat# ab9324, RRID:AB_307175) diluted 1:1000 (when counterstained with Kim-1); rabbit monoclonal anti-IL-6 (Thermo Fisher Scientific Cat# 416–7061-82, RRID:AB_3666112) diluted 1:400 (when counterstained with meprin β); rabbit monoclonal anti-PCNA (Cell Signaling Technology Cat# 13110, RRID:AB_2636979) diluted 1:4000; rabbit polyclonal anti-p-AKT (Cell Signaling Technology Cat# 9271, RRID:AB_329825) diluted 1:100 and rabbit polyclonal anti-p-ERK1/2 (Cell Signaling Technology Cat# 4370, RRID:AB_2315112) diluted 1:200. The slides were then rinsed three times in PBS for 5 minutes each and incubated for 1 h at room temperature in fluorophore-conjugated secondary antibodies diluted in the same dilution buffer: Alexa Fluor^®^ 647 (1:1000, Abcam, Cat# ab150075, RRID: AB_2752244) for rabbit polyclonal anti-KIM-1, rabbit monoclonal anti-IL-6, rabbit monoclonal anti-PCNA, rabbit polyclonal anti-p-AKT and rabbit polyclonal anti-p-ERK1/2; chicken monoclonal anti-mouse, Alexa Fluor^®^ 488 (1:1000, Invitrogen Cat# A-21200, RRID:AB_2535786) for mouse monoclonal anti-IL-6; Goat anti-Mouse IgG1 Cross-Adsorbed Secondary Antibody, Alexa Fluor^™^ 488 (1:3000, Thermo Fisher Scientific, Cat# A-21121, RRID AB_2535764) for mouse monoclonal anti-villin antibodies and chicken polyclonal anti-goat, Alexa Fluor^®^ 488 (1:1000, Invitrogen, Cat# A-21467, RRID:AB_141893) for goat polyclonal anti-mouse meprin β antibodies, chicken polyclonal anti-rabbit. Diluted 4, 6-diamidino-2-phenylindole (DAPI) (Vector Laboratories Cat# SK-4100, RRID: AB_2336382) was used for nuclear staining (1:1000). To prevent fluorescence signals from fading, all slides were covered by coverslips with prolong anti-fade reagent (Life Technologies, Carlsbad CA) and allowed to dry at room temperature overnight. Tissue sections were evaluated through a qualitative analysis to distinguish proximal tubules from distal tubules. The expression and localization of the evaluated proteins was done using a BZ-X700 Series all-in-one fluorescence microscope (KEYENCE Corporation of America, Elmwood, NJ) and imaged using BZ-X700 analysis software at 60 X magnification and the scale bar of 20 μm.

### Statistical analysis

Data analysis of mRNA expressions of the target genes was performed for each group versus WT control group. All data were analyzed by two-way ANOVA with Tukey’s pair-wise comparisons using GraphPad 7.0 Prism Software (GraphPad, La Jolla, CA). For mRNA expression data analysis, an unpaired *t* test was utilized to perform genes expression analysis for each group with WT control serving as the baseline control (n = 4 mice/group). Data for light microscopy utilized the calibrated ODs. Data of equal number of animals across all groups are presented as mean ± SEM. The p values ≤ 0.05 were considered statistically significant.

## Results

At 96 hours post-IR, elevated levels of the kidney injury biomarker KIM-1 were observed in select tubules for kidneys of both genotypes subjected to IR, but not in the counterpart control kidneys (Fig. 1A), thus confirming the induction of kidney injury. Immunofluorescence counterstaining with proximal tubule markers (meprin β in WT and villin in βKO kidneys) showed that increased KIM-1 expression was primarily in proximal tubules (PTs) and not distal tubules (DTs) (Fig. 1B). Interestingly, KIM-1 shedding into the PT lumen was detected in kidneys for both genotype, suggesting its excretion and clearance into the urine after kidney injury, as reported in previous AKI studies ^20,36,37^.

### Ischemia/reperfusion associated with increased IL-6 protein levels in kidney tissue at 96 h post-IR

**Figure 2:**
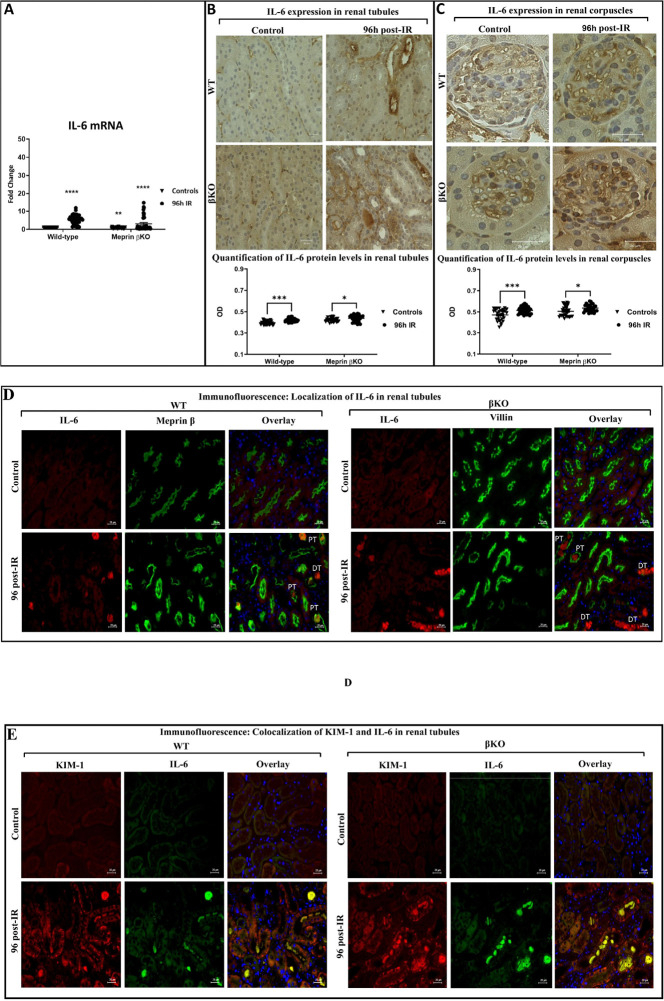
IL-6 mRNA and protein expression in kidney tissue from wild type (WT) and meprin β deficient mice (βKO) at 96 h post-IR. The real-time PCR data showed that mRNA expression levels of IL-6 increased in both genotypes subjected to IR **(A)**. Values for IL-6 mRNA levels were presented as fold change relative to control WT kidneys and normalized to GAPDH mRNA. Each value represents the mean ± SEM of triplicate combinations from 4 mice per group. Immunohistochemical staining for IL-6 in kidney tubules **(B)** and in renal corpuscles **(C)**. OD data were quantified using Image J analysis Software (ImageJ/Fiji 1.46) and analyzed by two-way ANOVA. Ten non-overlapping fields for tubular and 10 non-overlapping fields of renal corpuscle sections from each kidney were imaged at 60× magnification and the scale bar representing 20 μm. There was a significant increase in IL-6 mRNA and an increase in IL-6 protein expression in select tubules and in renal corpuscle at 96 h post- IR in both genotypes. Immunofluorescence counterstaining of IL-6 (red) in kidney tubules with the proximal tubule markers, meprin β (green) in wild-type (WT) and villin (green) in meprin β knockout (βKO) kidneys in renal tubules **(D)**. DAPI was used to stain the nuclei (blue). IL-6 expression was notably observed in the lumen of PTs and DTs in both genotype kidney sections at 96 h post-IR, suggesting IL-6 excretion and clearance into the urine at 96 h post-IR. Immunofluorescence counterstaining of IL-6 (green) and KIM-1 (red) in wild-type (WT) and meprin β knockout (βKO) kidneys **(E)**. DAPI was used to stain the nuclei (blue). Images at 60× magnification with a scale bar representing 20 μm. IL-6 expression was positively associated with KIM-1 within the same tubules, particularly in the lumen, indicating an association between IL-6 and KIM-1 in both genotypes subjected to IR. Data is expressed as mean ± SEM with P values as indicated, P ≤ 0.05 are considered statistically significant.

To determine the impact of meprin β expression/activity on IL-6 levels in vivo, mRNA and protein expression of IL-6 were evaluated in kidney tissue at 96 h-post-IR. RT-PCR data showed significant increases in mRNA levels of IL-6 in both WT (P ≤ 0.0001) and βKO mice (P ≤ 0.001) kidneys subjected to IR when compared to control kidneys at 96 h-post IR (Fig. 2A). Additionally, quantification of IL-6 staining intensity in kidney sections showed that significant increases in IL-6 expression in select renal tubules (Fig. 2B) for both WT (P ≤ 0.0001) and βKO mice (P ≤ 0.05) subjected to IR when compared to their respective control kidneys at 96 h-post IR. Similarly, IL-6 protein expression increased significantly in the renal corpuscles of kidney sections subjected to IR compared to their counterpart controls WT (P ≤ 0.0001) and βKO mice (P ≤ 0.05) (Fig. 2C). To identify the localization of increased IL-6 protein expression in kidney tubules, we used immunofluorescence counterstaining with proximal tubule biomarkers, meprin β in WT and villin in βKO kidneys. IL-6 expression was notably observed in PTs, which express meprin with some expression also detected in DTs, which lack meprins (Fig. 2D). We also observed increased IL-6 levels in the lumen of PTs and DTs in both genotype kidney sections at 96 h post-IR, suggesting IL-6 excretion and clearance into the urine at 96 h post-IR. We also used the immunofluorescence counterstaining to identify the co-localization of increased KIM-1 and IL-6 expression in kidney tissues, The data showed co-expression of KIM-1 and IL-6 within the same tubules, particularly in the lumen, indicating a positive association between IL-6 and KIM-1 (Fig. 2E). This suggests a tight connection between inflammation and tubular injury, with IL-6 signaling potentially driving kidney damage in tubules after 96 h of ischemia-reperfusion (IR) induction across both genotypes. The IL-6 protein staining intensity in renal corpuscles also increased significantly in both WT (P ≤ 0.0001) and βKO (P ≤ 0.01) at 96 h post-IR (Fig. 2C).

### Ischemia/reperfusion and meprin β expression associated with increased renal p-AKT levels at 96 h post-IR

**Figure 3:**
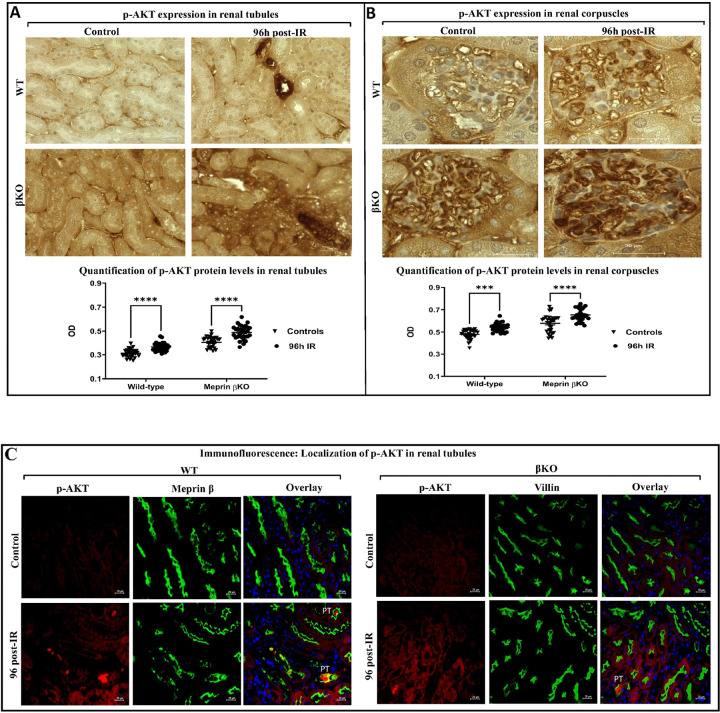
Protein expression of p-AKT in kidney tissue from wild type (WT) and meprin β deficient mice (βKO) at 96 h post-IR. Immunohistochemical staining for phosphorylated AKT protein in renal tubules in wild-type (WT) and meprin β knockout (βKO) kidneys **(A)** and renal corpuscles **(B)**. OD data were quantified using Image J analysis Software (ImageJ/Fiji 1.46) and analyzed by two-way ANOVA for 10 non-overlapping fields of tubular and 10 non-overlapping fields of renal corpuscle sections from each kidney and were imaged at 60× magnification with a scale bar representing 20 μm. There were significant increases in p-AKT levels in select tubules and in renal corpuscles of both genotypes’ kidneys subjected to IR. Immunofluorescence counterstaining of p-AKT (red) in kidney tubules in both genotypes **(C)**. Meprin β (green) and villin (green) were used as the proximal tubule biomarkers in WT and βKO, respectively. DAPI was used to stain the nuclei (blue). Images at 60× magnification with a scale bar representing 20 μm. Immunolocalization of p-AKT expression observed primarily in the lumen of PTs in both genotypes at 96 h post-IR. Data is expressed as mean ± SEM with P values as indicated, P ≤ 0.05 are considered statistically significant.

To determine whether meprin-β expression affects downstream modulators of the IL-6 signaling pathway, levels of phosphorylated Serine/Threonine Protein Kinase B, (PKB/AKT) on Serine 473 (p-AKT^Ser473^) protein were evaluated using a quantitative immunohistochemical staining approaches. Light microscopy and analysis of the immunostaining kidney sections showed that p-AKT protein levels significantly increased in select tubules of both genotypes’ kidney (P ≤ 0.0001) at 96 h following IR when compared to counterpart control kidneys (Fig. 3A). The staining intensity for p-AKT in renal corpuscles showed a similar significant increase pattern as in renal tubules of WT (P ≤ 0.001) and βKO (P ≤ 0.0001) at 96 h post-IR (Fig. 3B). To determine whether meprin expression correlates with p-AKT expression in mice kidney sections, we used immunofluorescent counterstaining with proximal tubule (PT) markers (meprin β for WT and villin for βKO, respectively). Our data showed that at 96 hours post-IR, p-AKT expressions were primarily observed in meprin β-expressing proximal tubules (PTs), compared to distal kidney tubules (DTs), which do not express meprin β (Fig. 3C). Additionally, immunofluorescence staining showed p-AKT expression in the lumen of PTs of both genotypes’ kidney sections at 96 h post-IR, suggesting release p-AKT into filtration and subsequently into urine.

### Meprin β deficiency associated with increase p-ERK1/2 protein levels in kidney tissue at 96 h post-IR

**Figure 4:**
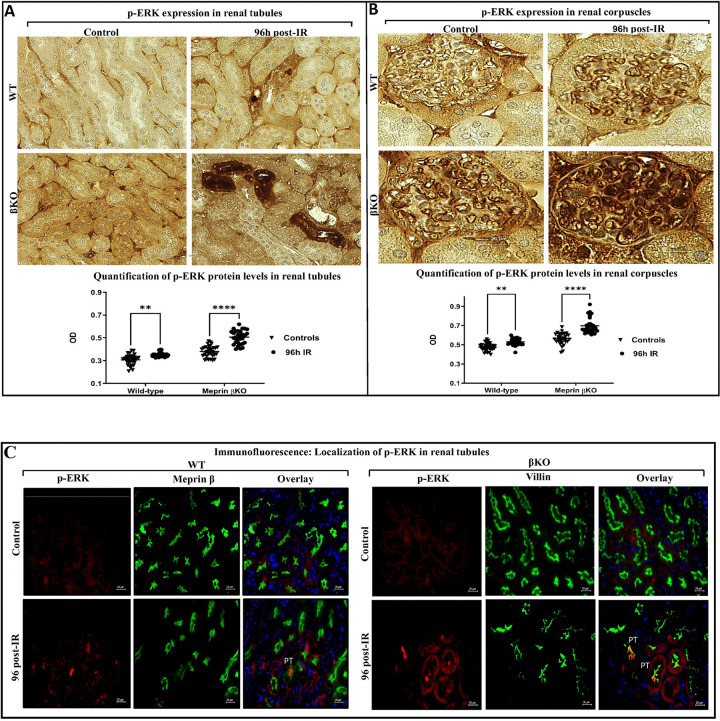
Protein expression of p-ERK in kidney tissue from wild type (WT) and meprin β deficient mice (βKO) at 96 h post-IR. Immunohistochemical staining for phosphorylated ERK in WT and meprin βKO kidney tubules **(A)**. Immunohistochemical staining for p-ERK in renal corpuscles of WT and meprin βKO mice kidneys **(B)**. OD data were quantified using Image J analysis Software (ImageJ/Fiji 1.46) and analyzed by two-way ANOVA for 10 non-overlapping fields from renal tubular and renal corpuscle sections from each kidney. Images at 60× magnification and the scale bar representing 20 μm. There were significant increases in p-ERK levels in select tubules and in renal corpuscles of both genotypes 96 h post-IR. Immunofluorescence counterstaining of p-ERK (red) in kidney tubules with the proximal tubule markers, meprin β (green) in wild-type (WT) and villin (green) in meprin β knockout (βKO) kidneys in renal tubules **(C)**. DAPI was used to stain the nuclei (blue). Images at 60× magnification with a scale bar representing 20 μm. Protein expression of p-EKT was observed in the lumen of PTs only in both genotypes’ kidneys subjected to IR. Data is expressed as mean ± SEM with P values as indicated, P ≤ 0.05 are considered statistically significant.

To assess whether meprin β expression influences additional downstream modulators of the IL-6 signaling pathway, we evaluated phosphorylated Mitogen-Activated Protein Kinases (MAPKs) Phospho-p44/42 MAPK (Erk1/2) (Thr202/Tyr204). The antibodies used to detect the endogenous levels of p44 and p42 MAP Kinase (Erk1 and Erk2) which are either dually phosphorylated at Thr202 and Tyr204 of Erk1 (Thr185 and Tyr187 of Erk2) or singly phosphorylated at Thr202. Immunohistochemical staining coupled with evaluation by light microscopy were used to evaluate the levels of p-ERK. Quantification of the immunostaining intensity showed that the levels of p-ERK proteins were significantly increased in select tubules in WT (P ≤ 0.01) and βKO (P ≤ 0.0001) kidneys after 96 h post-IR when compared to their control counterparts (Fig. 4A). Similar significant increases of the staining intensity for p-ERK were demonstrated in renal corpuscles in both genotypes subjected to IR (Fig. 4B). Immunofluorescence counterstaining method was used to determine the localization of the p-ERK1/2 expression in the kidney tubules (Fig. 4C). Like p-AKT, p-ERK1/2 expression was mainly observed in PTs of the kidney tissues of WT and βKO compared to DTs at 96 h post-IR. Also, accumulation of p-ERK1/2 protein was observed in the lumen of the PTs only of both genotype kidney sections subjected to IR, implying that p-ERK1/2 is discharged into the filtration process and subsequently excreted through urine.

### Meprin β deficiency associated with mediators of cellular proliferation in renal corpuscles at 96 h post-IR

**Figure 5:**
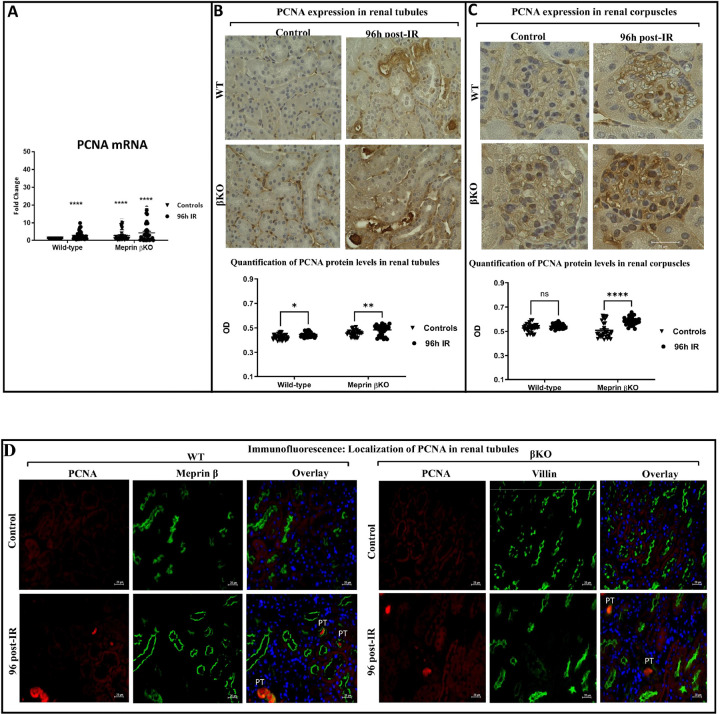
PCNA protein expression in kidney tissue from wild type (WT) and meprin β deficient mice (βKO) at 96 h post-IR. The real-time PCR data showed that mRNA expression levels of PCNA increased in both genotypes subjected to IR **(A)**. Values for PCNA mRNA levels were presented as fold change relative to control WT kidneys and normalized to GAPDH mRNA. Each value represents the mean ± SEM of triplicate combinations from 4 mice per group. Immunohistochemical staining for PCNA in kidney tubules in wild-type (WT) and meprin β knockout (βKO) kidneys **(B)**. Immunostaining for PCNA in renal corpuscles in both genotypes **(C)**. OD data were quantified using Image J analysis Software (ImageJ/Fiji 1.46) and analyzed by two-way ANOVA for 10 non-overlapping fields from renal tubular and renal corpuscle sections from each kidney. Images at 60× magnification and the scale bar representing 20 μm. There was a significant increase in PCNA protein expression in select tubules in both genotypes, but only in βKO in renal corpuscle of kidneys subjected to IR. Immunofluorescence counterstaining of PCNA (red) in kidney tubules with the proximal tubule markers, meprin β (green) in wild-type (WT) and villin (green) in meprin β knockout (βKO) kidneysin renal tubules **(D)**. DAPI was used to stain the nuclei (blue). Images at 60× magnification with a scale bar representing 20 μm. PCNA was primarily expressed in the lumen of PTs in both genotype kidney sections at 96 h post-IR. Data is expressed as mean ± SEM with P values as indicated, P ≤ 0.05 are considered statistically significant.

To determine whether meprin β expression impacts downstream cellular proliferation of the IL-6 signaling pathway at 96h post-IR (considered the repair phase level), mRNA and protein levels of cellular proliferation marker (proliferating nuclear antigen cell, PCNA) were evaluated in kidney tissue. The data showed that mRNA levels of PCNA significantly increased in kidneys subjected to IR for both genotypes when compared to control mice (P < 0.01) at 96 h-post IR (Fig. 5A). Increased PCNA protein levels were confirmed using light microscopy, with a quantitative immunostaining intensity method for PCNA. Immunohistochemistry data showed that PCNA significantly increased (P < 0.05) in specific tubules of both genotypes for kidneys at 96 h post-IR when compared to control kidneys (Fig. 5B). When compared to βKO kidneys, the PCNA baseline levels were lower in WT kidneys (P < 0.01). Interestingly, the immunohistochemical analysis showed that the staining intensity for PCNA in renal corpuscles significantly increased in βKO only (P ≤ 0.0001) and not in WT at 96 h post-IR (Fig. 5C). Immunofluorescence counterstaining of PCNA with proximal tubule markers (meprin β in WT and villin in βKO) showed expression of PCNA mainly in PTs of both genotypes’ kidneys subjected to IR (Fig. 5D). Additionally, PCNA levels were observed in the lumen of PTs in both genotypes at 96 h post-IR, indicating discharge PCNA into the filtration process and subsequently passing into the urine.

## Discussion

Ischemia-reperfusion (IR) is a major contributor to acute kidney injury (AKI), a condition associated with high morbidity and mortality rates ^1,2^. Several studies have shown that inflammation is a key mechanism in the progression of AKI induced by IR ^3,4,7^. At the initial stages of inflammation, circulating monocytes are recruited to the injury site, where they differentiate into classically activated M1 macrophages ^8^. These macrophages release pro-inflammatory cytokines, including Interleukin-6 (IL-6), as part of a process that promotes cell proliferation and survival during immune responses to cellular stress ^38,39^. When IL-6 is bound to its soluble receptor (sIL-6R), it activates the IL-6 trans-signaling pathway, an anti-inflammatory process in IR-induced AKI ^9,40–45^, promoting repair processes. Meprin metalloproteinases are implicated in the pathophysiology of kidney injury and play a critical role in inflammation, in part by processing and inactivating IL-6 ^14,46^. Meprins are abundantly expressed in the brush-border membrane (BBM) of kidney proximal tubules. Interestingly, meprins undergo redistribution from BBMs to the cytoplasm and basolateral compartments of proximal tubule cells in IR-induced AKI ^47^. This redistribution brings meprin β in direct contact with extracellular matrix (ECM) in the basal lamina and enhances their accessibility for proteolytic cleavage ^48^. Meprin β modulates IL-6 activity by inactivating it through proteolytic processing ^14^. It also cleaves the membrane-bound IL-6 receptor (mbIL-6R), enabling classical IL-6 signaling in cells expressing mbIL-6R ^49^. In contrast, the release of the soluble IL-6 receptor (sIL-6R) activates trans-signaling in cells lacking mbIL-6R, such as proximal tubule epithelial cells and macrophages ^12,50^. Data from the current study revealed an increase in IL-6 mRNA expression in both genotypes at 96 h post-IR. Additionally, immunohistochemical staining showed that IL-6 protein expression increased in select renal tubules and in renal corpuscles of both genotypes. These data align with our previous findings at 24 hours post-IR ^20^, which is the early phase of kidney injury. The high levels of IL-6 at 96 h post-IR, considered a reparative phase of AKI, suggests that IL-6 plays a role not only in the injury phase but also in the recovery phase of the inflammatory response. Immunofluorescence counterstaining with proximal tubule biomarkers showed that increased IL-6 expression occurred in both proximal tubules (PTs), which express meprin β, with some expression detected in distal tubules (DTs), which lack meprin β. We also observed increases in IL-6 levels in the lumen of both PTs and DTs in WT and βKO kidneys at 96 h post-IR. This suggests a release of IL-6 into filtrate and subsequently into urine as supported by previous studies ^47,51^. Additionally, counterstaining of IL-6 and kidney injury marker (KIM-1) in the present study showed that IL-6 expression associated with kidney injury in proximal tubules in both genotypes up to 96 h post-IR, which was previously seen at the acute phase ^20^. These findings suggest a dual role for IL-6 in both inflammation and tissue repair despite the ongoing processing by meprin β. When IL-6 binds to its receptor, forming the IL-6/IL6R complex and subsequently interacts with the membrane-bound gp130 dimer, it triggers the activation and phosphorylation of Janus Kinase-Signal Transducer and Activator of Transcription (JAK/STAT), Mitogen-Activated Protein Kinase (MAPK), and Phosphatidylinositol 3-Kinase/Akt Kinase (PI3K/AKT) pathways ^52^. These activated pathways are known to regulate various downstream processes including cellular apoptosis, survival, and proliferation ^28^. Activation of the trans-signaling IL-6 has been demonstrated to enhance cellular proliferation ^15,17^. This activation of the cellular proliferation involves MAPK/ERK signaling pathway ^21,22^, the PI3K/AKT pathway ^23–25^, or their crosstalk AKT/ERK ^27–29^. To date, the mechanism by which meprin β regulates IL-6-induced cellular proliferation remains unclear. It is not yet known whether this regulation occurs through the MAPK/ERK pathway, the PI3K/AKT pathway, or their crosstalk (AKT/ERK). Therefore, the current study investigated the effect of meprin β expression on the expression levels of the two main downstream modulators of proliferation involving the IL-6 signaling pathway, AKT and ERK. Our data demonstrated a significant increase in phosphorylated forms of both meditators (p-AKT and p-ERK) in select tubules and in renal corpuscles at 96 hours post-IR, suggesting activation of the AKT/ERK pathway. Previous studies showed that mitochondrial AKT activation plays a role in preventing the progression of chronic kidney disease via attenuating oxidative stress, improving mitochondrial function, and reducing tubular injury ^53,54^. AKT was also shown to play a pivotal role in the pathogenesis of renal cell proliferation ^55^. These findings suggest a critical role for the IL-6 in activating the AKT pathway during kidney recovery, potentially driving essential repair mechanisms and contributing to cellular proliferation. Additionally, the observed increase in p-ERK levels could be due to the role of ERK activation in ischemia-reperfusion injury, frequently mediated by reactive oxygen species (ROS) ^56,57^. These ROS-dependent pathways are shown to be essential for ERK signaling driving cell survival and repair processes. ERK1/2 is a conserved family of serine/threonine kinases involved in cell proliferation and differentiation ^58,59^. This pathway is activated through various extracellular stimuli, including mitogens, growth factors, and cytokines including IL-6 ^60–62^. The activation of ERK pathway plays a critical role in the proliferation and repair of renal tubular epithelial cells which helps facilitate the restoration of damaged tubules and preventing fibrosis progression ^56^. Previous study findings from our group are aligned with the current data, indicating substantial increases in p-ERK levels in both meprin β knockout (βKO) and wild-type (WT) at 6 hours post-IR ^33^. These findings suggest that the increased p-ERK levels at 96 h post-IR may be due to its sustained activity, supporting tissue repair and functional recovery in IR-induced kidney injury. The PI3K/AKT/IL-6 pathway plays a pivotal role in cancer cell proliferation and survival ^63^. On the other hand, the IL-6-/ERK pathway was shown to be one of the key activators of cell proliferation, cancer cell growth, and tumor progression ^64,65^. Interestingly, both AKT and ERK signaling pathways are activated in renal tubules and renal corpuscles for up to 96 hours following IR-induced acute kidney injury (AKI). These intriguing observations, coupled with the role of AKT and ERK as crucial intracellular signaling pathways for cellular proliferation, prompted us to investigate whether the IL-6/AKT/ERK axis influences cell proliferation in relation to meprin activity during the repair phase of IR injury at 96 hours post-IR. To explore this, we examined Proliferating Cell Nuclear Antigen (PCNA), a well-established marker of cellular proliferation, which is known to be induced by the activation of IL-6 trans-signaling. PCNA is prominently expressed in the S3 segment of the proximal tubule following ischemic injury indicating its role in kidney repair ^66,67^. However, the mechanisms linking meprin β regulation of IL-6 trans-signaling to this proliferation remain unclear. Our RT-PCR data revealed significant increase of PCNA mRNA expression levels in both genotypes at 96 h post-IR. This was further supported by quantitative immunohistochemical analysis, which demonstrated a substantial increase in PCNA protein expression in specific tubules, suggesting the activation of mitogenic responses essential for tubular repair and regeneration phase ^68,69^. Interestingly, our results demonstrated a significant increase in PCNA protein expression in renal corpuscles of βKO mice, but not in WT, at 96 h post-IR, suggesting a unique proliferative response in the absence of meprin β expression and potential role of meprin β acts as a negative regulator of cell proliferation in the renal corpuscles. Interestingly, PCNA was detected in the lumen exclusively within proximal tubules, suggesting shedding into the urinary tract. Consistent with our findings, PCNA excretion in urine was previously reported ^70^, emphasizing its potential as a valuable non-invasive biomarker for clinical assessments. Furthermore, as proximal tubules are known to be more susceptible to damage due to their high metabolic activity and critical role in filtration and reabsorption ^71^, the exclusive detection of PCNA, along with p-AKT and p-ERK, in the lumen of PTs likely reflects proliferative and repair responses to injury-induced stress mediated by the AKT/ERK signaling pathway during the recovery phase (96 hours post-IR). Collectively, our findings suggest that meprin may regulate cellular proliferation through IL-6-mediated activation of the AKT/ERK signaling pathway, promoting the expression of proliferation proteins such as PCNA and facilitating tissue repair during the kidney recovery phase.

## Conclusion

Taking together, these findings highlight the roles of IL-6 and meprin β in modulating AKT/ERK-mediated cellular proliferation, emphasizing their complex, tissue-specific functions in kidney injury and repair. Their involvement in conditions characterized by dysregulated proliferation, such as IR injury and proliferative kidney diseases, underscores their therapeutic potential. While our results suggest that meprin β regulates cellular proliferation through the IL-6-mediated AKT/ERK signaling pathway during the repair phase of IR-induced kidney injury, further studies are needed to elucidate this interplay and its role in recovery. Targeting PCNA and meprin β could offer therapeutic strategies to limit tumor growth and inflammation-driven progression in kidney injury and cancer.

## Data Availability

The datasets used and analyzed during the current study are available from the corresponding author upon reasonable request.

## Abbreviations

2-ΔΔCt method: 2 (-Delta Delta C(T)) Method
AKI: Acute Kidney Injury
AKT: Protein kinase B
BBM: Brush-Border Membrane
Bcl-2: B-Cell Lymphoma/Leukemia 2
cDNA: Complementary Deoxyribonucleic Acid
DAPI: 4,6-Diamidino-2-Phenylindole
DTs: Distal Tubules
ECM: Extracellular Matrix
ERK: Extracellular signal-Regulated Kinase
FC: Fold Change
GAPDH: Glyceraldehyde-3-Phosphate Dehydrogenase
IACUC: Institutional Animal Care and Use Committee
IL-6: Interleukin 6
IR: Ischemia/Reperfusion
JAK: Janus Kinase
KIM-1: Kidney Injury Molecule-1
LARU: Laboratory Animal Resource Unit of North Carolina
MAPK: Mitogen-activated protein kinase
mbIL-6R: Membrane-bound Interleukin 6 Receptor
Meprin A: Meprin A is a Homooligomer of α Subunits (α-α) or a Heterooligomer of α and β Subunits (α-β)
Meprin B: Meprin B is a Homooligomer of β Subunits (β-β)
mRNA: Messenger Ribonucleic Acid
PBS: Phosphate Buffer Saline
PCNA: Proliferating Cell Nuclear Antigen
PCR: Polymerase Chain Reaction
p-ERK1/2: Phosphorylated Extracellular Signal-Regulated Kinase 1/2
PI3K: Phosphatidylinositol 3-Kinase/Akt kinase
PKB/AKT: Phosphorylated Serine/Threonine Protein Kinase B
PTs: Proximal Tubules
RNA: Ribonucleic Acid
ROS: Reactive Oxygen Species
sIL-6R: Soluble Interleukin 6 Receptor
STAT: Signal Transducer and Activator of Transcription
WT: Wild-Type
βKO: Meprin-β Knockout Mice, Deficient in Meprin B (β-β) and the Heterodimeric Form of Meprin A (α-β)
